# HSPA12A targets the cytoplasmic domain and affects the trafficking of the Amyloid Precursor Protein receptor SorLA

**DOI:** 10.1038/s41598-018-37336-6

**Published:** 2019-01-24

**Authors:** Peder Madsen, Toke Jost Isaksen, Piotr Siupka, Andrea E. Tóth, Mette Nyegaard, Camilla Gustafsen, Morten S. Nielsen

**Affiliations:** Department of Biomedicine, Aarhus University, 8000 Aarhus, Denmark

## Abstract

SorLA and Sortilin are multifunctional receptors involved in endocytosis and intracellular sorting of different and unrelated ligands. SorLA has recently attracted much attention as a novel strong risk gene for Alzheimer’s disease, and much effort is currently being put into understanding the underlying molecular mechanism. Trafficking of SorLA and Sortilin are mediated by interacting with AP-1, AP-2, GGA 1-3 and the retromer complex. Although these cytosolic adaptor proteins all bind to both SorLA and Sortilin, a large fraction of intracellular Sortilin and SorLA are located in different subcellular vesicles. This indicates that unknown specialised adaptor proteins targeting SorLA for trafficking are yet to be discovered. We have identified HSPA12A as a new adaptor protein that, among Vps10p-D receptors, selectively binds to SorLA in an ADP/ATP dependent manner. This is the first described substrate of HSPA12A, and we demonstrate that the binding, which affects both endocytic speed and subcellular localisation of SorLA, is mediated by specific acidic residues in the cytosolic domain of SorLA. The identification of the relatively unknown HSPA12A as a SorLA specific interaction partner could lead to novel insight into the molecular mechanism of SorLA, and re-emphasises the role of heat shock proteins in neurodegenerative diseases.

## Introduction

SorLA is a multifunctional receptor involved in endocytosis and intracellular sorting of different and unrelated ligands. SorLA is a member of the vacuolar protein sorting 10 domain receptor family (Vps10p-D) of type 1 receptors, comprising also Sortilin and SorCS1-3^1,2^. The family is characterised by an N-terminal Vps10p domain, a unique 10-bladed β-propeller domain with ligand binding capacity^3^, and a short cytoplasmic domain (cd) that mediate cellular trafficking through interaction with cytosolic adaptor proteins.

The extracellular domain of SorLA interacts with peptide ligands such as apolipoprotein E (apoE), glia cell line-derived neurotrophic factor (GDNF), lipoprotein lipase, as well as its own propeptide^4–6^. SorLA also modulates Cytokine-Like Factor-1:Cardiotrophin-like Cytokine (CLC:CLF-1) signalling through the receptor complex consisting of the ciliary neurotrophic factor receptor (CNTFR) and the gp130/leukemia inhibitory factor receptor (LIFR)^4^. The cd mediates endocytosis and uptake of ligands bound at the surface membrane, as well as transport between trans-Golgi compartments and endosomes^7^. SorLA is furthermore localised in a polarised manner in early basolateral sorting endosomes and at the basolateral membrane of epithelial MDCK cells, and in the somatodendritic area of hippocampal neurons^8^. The cd contains several motifs for binding of cytoplasmic adaptor proteins (e.g., Adaptor protein complexes-1 and -2 (AP), Golgi-localising, Gamma-adaptin ear domain homology, ARF-binding proteins 1 to 3 (Golgi-localising, Gamma-adaptin ear domain homology, ARF-binding proteins) and elements of the retromer complex) involved in receptor trafficking^7,8^. While these cytosolic adaptor proteins all bind to both SorLA and Sortilin, a large fraction of intracellular Sortilin and SorLA are located in different subcellular vesicles, indicating that more yet unrecognised adaptor proteins are involved in the trafficking and localisation of SorLA and Sortilin^7^.

SorLA is encoded by the *SORL1* gene, which has recently been established as a strong risk gene for early onset of Alzheimer’s disease both in family and case control studies^9–15^, with loss of function variants, in particular, segregating with disease in families^13^ and found in single cases in case control studies^16^. The mechanism is believed to be through SorLA’s role in processing amyloid precursor protein (APP) and the generation of Aβ-amyloid peptide^17^, although the mechanism is not entirely clear.

Heat shock proteins (HSPs) are a diverse group of proteins characterised by being up-regulated under stressful conditions^18^. The members exhibit molecular chaperone activity by binding to newly synthesised proteins thereby catalysing correct folding, or by mediating refolding of damaged proteins. Molecular chaperones thereby provide a first-line of defence against misfolded, aggregation-prone proteins, and are among the most potent suppressors of neurodegeneration in animal models of human disease^19,20^. The HSP molecular chaperones are subdivided in groups based on their molecular mass: HSPH (HSP110), HSPC (HSP90), HSPA (HSP70), DNAJ (HSP40) and HSPB (small HSP)^21^. The HSP70 protein family, encoded by 17 genes^22^, is mainly involved in ATP-driven refolding and solubilisation of aggregated proteins^20^. HSP70s display a common domain structure composed of a 44-kDa N-terminal nucleotide binding domain (NBD) that binds and hydrolyses ATP, a middle domain with protease sensitive sites, and a 28-kDa C-terminal substrate binding domain (SBD) that binds extended polypeptides^23^. The NBD is conserved in all of the HSP70 family members, with the exception of the two *HSPA12* genes encoding divergent NBDs with uncharacterised nucleotide binding properties^24^. The activities of HSP70s depend on their ATP-regulated ability to interact with exposed hydrophobic surfaces of proteins. ATP hydrolysis and ADP/ATP exchange are key events for substrate binding and HSP70 release during folding^25^ of nascent polypeptides. The proteins HSPA12A and HSPA12B are distant members of the HSP70 family mainly due to an atypical ATP-binding domain^24^. Like SorLA, HSP70 proteins also play an important role in neurodegenerative diseases, where they are involved in preventing protein misfolding and inhibiting aggregation.

In this study, we set out to identify novel adaptor proteins to the cd of SorLA, exclusively targeting and determining the fate of SorLA. Using a Yeast-Two-Hybrid (Y2H) screening, HSPA12A was identified as a novel adaptor protein for SorLA, delaying internalisation of SorLA and when binding, leading to relocation of subcellular SorLA-positive vesicles.

## Results

### Initial screen for adaptor proteins using Y2H

**Figure 1:**
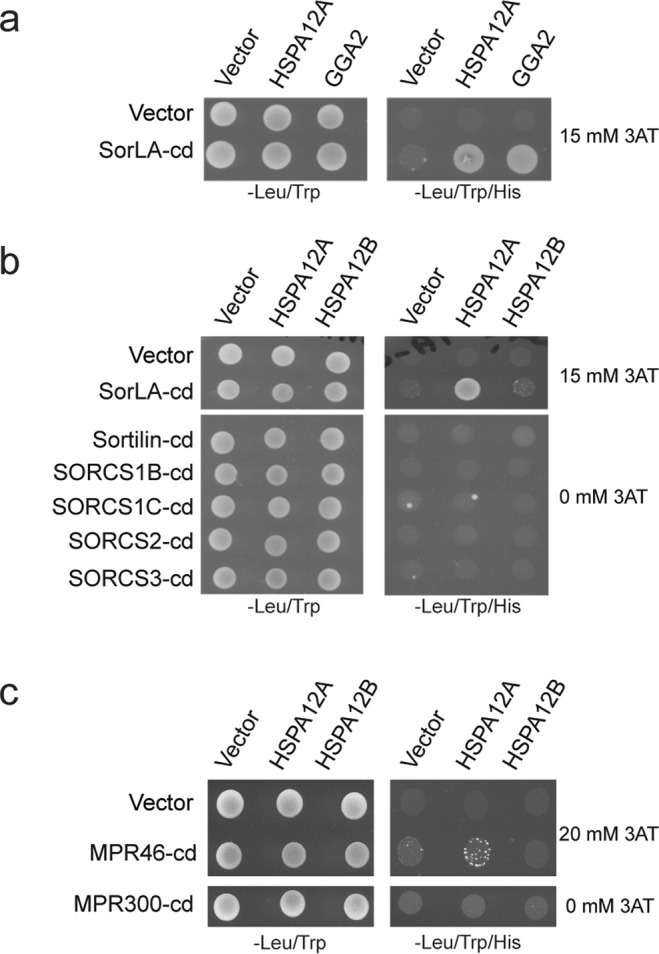
HSPA12A interacts with the cd of SorLA. (**a**) Y2H assay with the SorLA-cd against the substrate binding domains of HSPA12A and GGA2. (**b,c**) Y2H assay with the related cd of all Vsp10p receptors and the two mannose 6-phosphate receptors (MPR-46 and -300) performed against the substrate binding domains of HSPA12A and HSPA12B. 3-aminotriazole (3AT) was used to suppress bait auto-activation of SorLA-cd and MPR-cds. All Y2H were done three times.

To identify novel adaptor proteins targeting the cd of SorLA, a commercial Y2H screen was performed by Hybrigenics (Paris, France, http://www.hybrigenics.com). An adult human brain random-primed cDNA library, transformed into the Y187 yeast strain, was used for mating. Among resulting clones, several encoded a C-terminal fragment of HSPA12A. In addition, several clones encoding a VPS-27/Hrs/STAM domain (VHS) from either GGA-1 or -2 were identified, which previously have been described as ligands to the cd’s of SorLA and Sortilin^26,27^. To certify data from the primary screening, library and bait plasmids were purchased from Hybrigenics for additional verification using control plasmids without inserts. The SorLA cd (cd) elicited a positive signal in the presence of the 163 C-terminal amino acids from the HSPA12A protein and from the GGA2-VHS domain; neither bait nor prey plasmids exhibited auto-reactivity under the test conditions (Fig. 1a). Auto-reactivity from the SorLA-cd was suppressed by addition of 15 mM 3-AT. Testing HSPA12A and GGA2-VHS against the Sortilin-cd produced only a positive signal from the GGA2-VHS peptide (not shown). Testing the corresponding C-terminal fragment from the closely related HSPA12B^24^ against the SorLA-cd was also negative (Fig. 1b). In addition, cd’s from the remaining Vps10p-D receptors (Sortilin and SorCS1-3), which have a highly overlapping sorting pattern, were tested against the HSPA12-A and -B with a negative outcome (Fig. 1b). The two mannose phosphate receptors (MPR-46 and -300) mediate sorting of lysosomal hydrolases by adaptor proteins binding to the cd’s. Since MPR46 and MPR300 are shuttled between trans-Golgi network (TGN) and endosomes by the same mechanism as Sortilin and SorLA, the cd’s of both receptors were tested in Y2H against both HSPA12-A and -B. Auto-reactivity from the MPR46-cd was suppressed by addition of 20 mM 3-AT. The result was negative for the MPR300-cd, however a weak interaction with MPR46-cd cannot be excluded (Fig. 1c). APP is a substrate for BACE-1, a beta-secretase involved in the generation of amyloid-β, a hallmark of Alzheimer’s disease^28,29^. BACE-1 and APP are type 1 receptors with short cd’s, and both are potential ligands for HSPA12A. Testing the cd’s of BACE-1 and APP were unresponsive for interaction with HSPA12-A and -B (not shown).

### Confirmation of HSPA12A using pull-down analysis

**Figure 2:**
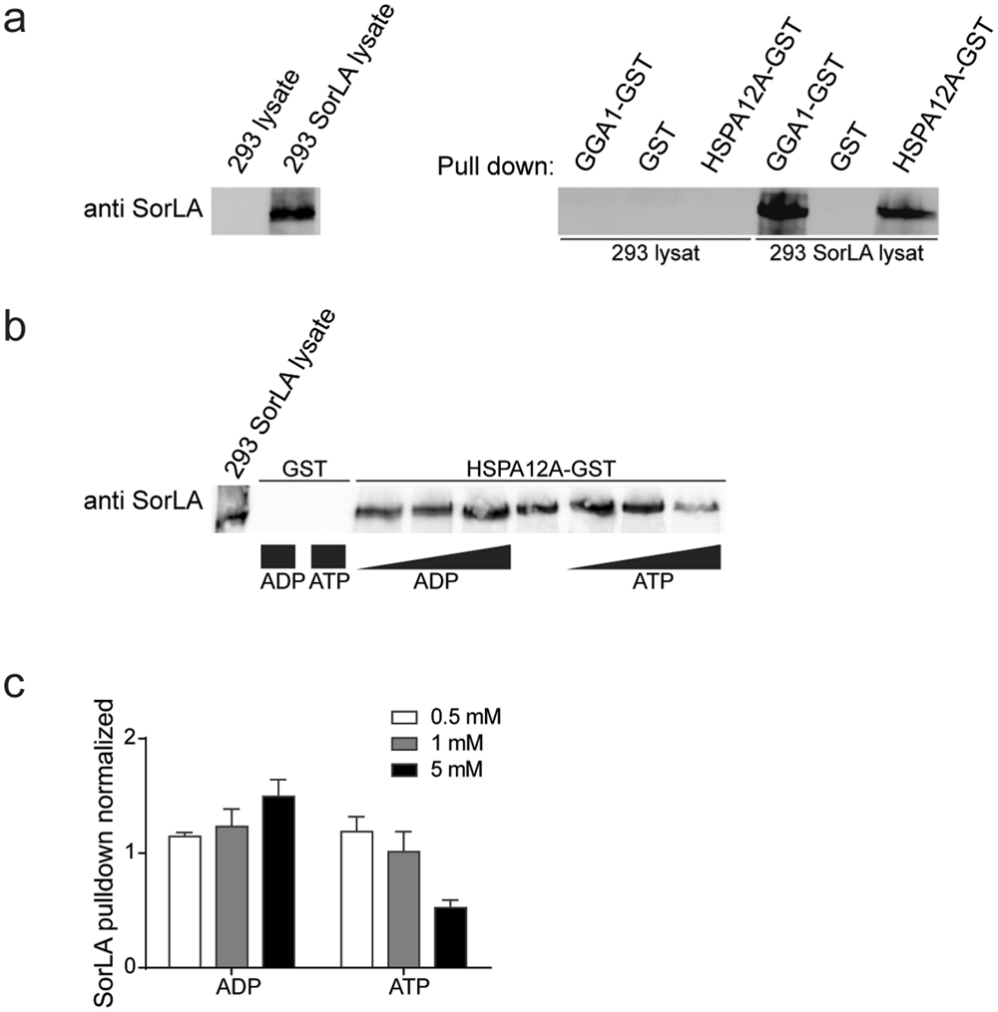
HSPA12A and SorLA interaction depends on ATP. (**a**) Pull-down of SorLA using GGA1-GST, GST or HSPA12A-GST fusion proteins pre-immobilised on glutathione-beads. Precipitates were analysed by Western blotting using an antibody against SorLA. The blot on the left side demonstrate the presence of SorLA in input lysates. Pull-down was confirmed in three independent trails. (**b**) Pull-down of SorLA from transfected 293 cells using GST (lane 2 and 3 from the left side) or HSPA12A-GST fusion protein. ATP or ADP was added to final concentrations of 0.5, 1.0 or 5.0 mM as indicated by the increasing black bar during incubation period. The first lane demonstrate the presence of SorLA in the input lysate (n = 3). (**c**) Average (±SEM) SorLA pull-down with 0.5, 1.0 or 5.0 mM ATP or ADP added. Average values (n = 3) are from three independent pull-down experiments, and have been normalised to pull-down of SorLA in each experiment without added ATP or ADP. The Western blots have been cropped to reduce the size of the figure. The entire membranes with maker size and without contrast adjustments, can be inspected in the Supplemental Materials.

Pull-down assays were performed to verify the interaction between the cd of SorLA and HSPA12A. Full-length HSPA12A and the VHS domain of GGA1, both N-terminal fused to glutathione-S-transferase (GST), were expressed in *E. coli* and purified on glutathione-beads. Full-length SorLA was stably expressed in HEK293 cells (Fig. 2a). Lysate from HEK293 cells expressing SorLA was incubated with pre-immobilised GST fusion proteins or GST overnight at 4 °C. Analysis by western blotting established that both HSPA12A and GGA1 fusion proteins avidly precipitate SorLA from the HEK293 lysates, in contrast to the unresponsive outcome from using GST. Untransfected HEK293 cells were used as negative control (Fig. 2a). HSP70 proteins exist in two conformational forms, bound either with ATP or ADP at the N-terminal ATPase domain^23^. The ADP bound conformation exhibits elevated substrate binding activity^19^. The impact of ATP and ADP on HSPA12A binding to SorLA was assessed by GST pull-down assay in the presence of 0.5 mM, 1 mM and 5 mM ATP and ADP (0.5 mM ADP vs 5 mM ADP, p = 0.0178; 0.5 mM ATP vs 5 mM ATP, p = 0.001; One-way ANOVA followed by post-hoc Tukey multiple comparison test) (Fig. 2b,c). Increasing ADP levels induce precipitation of SorLA, whereas increasing ATP levels significantly decrease precipitation of SorLA. Control pull-down with GST, in the presence of added ATP or ADP, did not precipitate SorLA (Fig. 2b). Hence, the interaction between HSPA12A and SorLA is regulated by adenonucleotides bound to the N-terminal ATPase domain of HSPA12A.

### HSPA12A binding is determined by C-terminal acidic residues in the SorLA-cd

**Figure 3:**
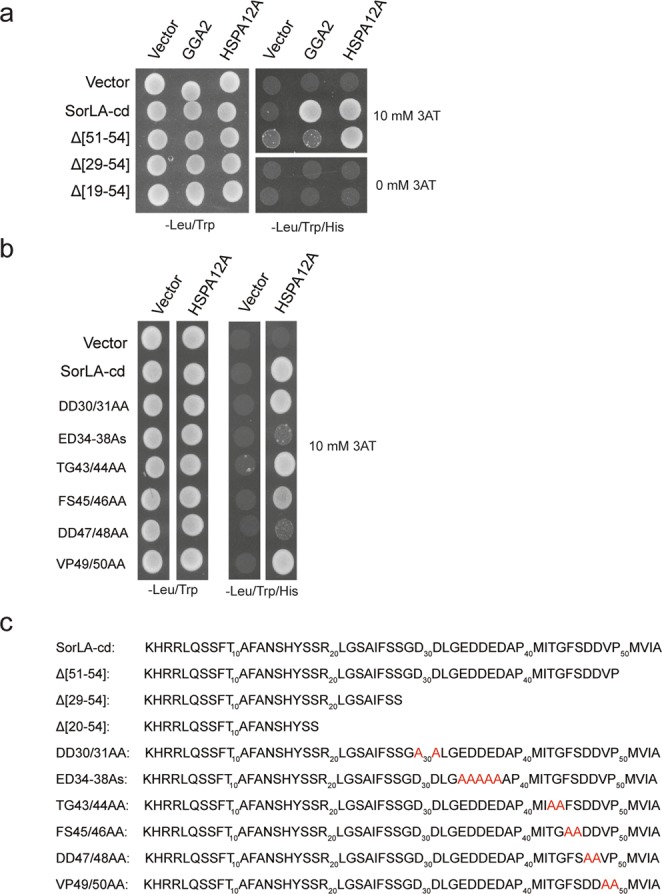
HSPA12A binds SorLA-cd via C-terminal acidic residues. (**a,b**) Y2H assay with truncated and point mutated SorLA-cd tested against the substrate binding domains of HSPA12A and GGA2. (**c**) Amino acid sequence of SorLA-cd variants used for Y2H assay. All Y2H were done three times.

To identify the amino acids in the SorLA-cd contributing to HSPA12A binding, C-terminal truncated mutants were tested for interaction by Y2H using the HSPA12A clone identified by Hybrigenics. Auto-reactivity from the SorLA-cd was suppressed by addition of 10 mM 3-AT Deletion of the 4 C-terminal amino acids (Δ51–54) retained the signal from HSPA12A, but prohibited interaction with GGA2-VHS (Fig. 3a) due to partial loss of the recognition site for the VHS domain^7^. The truncated constructs Δ29–54 and Δ20–54 display negative signals for both HSPA12A and GGA2-VHS (Fig. 3a), suggesting that the acidic-rich amino acid region, from the glycine residue on position 29 to the proline on position 50, in the SorLA-cd is critical for HSPA12A binding (Fig. 3a). Acidic clusters (AC) play a demonstrated role as intracellular sorting signals for endocytic receptors^7,30,31^. Consequently, the pentameric AC (E34D35D36E37D38) and the two di-ACs (D31D32; D47D48) were independently mutated to alanine residues (Fig. 3c), and additional amino acids near the C-terminal were independently mutated to alanine residues (Fig. 3c). The Y2H signals from HSPA12A were strongly repressed by the pentameric AC and the C-terminal di-aspartic cluster (D47D48) mutants; the remaining mutants exhibited a response equal to the wild-type SorLA-cd. Accordingly, it is concluded that the 2 C-terminal acidic clusters both contribute to the binding between the SorLA-cd and HSPA12A (Fig. 3c). Thus, HSPA12A and GGA2 are sharing a C-terminal overlapping AC (D47D48) binding motif^27^.

### Co-localisation of HSPA12A and SorLA

**Figure 4:**
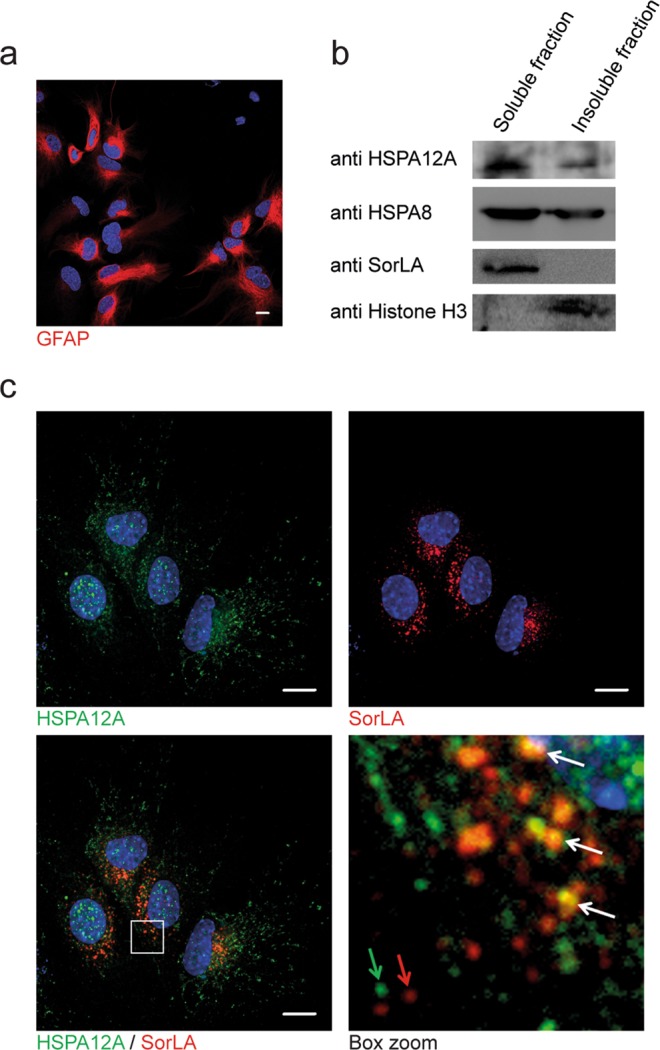
HSPA12A and SorLA co-express and co-localise in cortical astrocytes. (**a**) Cortical astrocyte cultures were prepared from p0 C57BL/6 wild-type mice, and purity of cultures were tested by immunofluorescence microscopy of GFAP (red) and Hoechst (blue) for nuclei stain. (**b**) Western blot analysis of fractionated lysate from cultured cortical astrocytes showing expression of both SorLA and HSPA12A. (**c**) Immunofluorescence microscopy of HSPA12A (green) and SorLA (red). The white box in the merge picture is magnified and presented (box zoom). White arrows point to perinuclear vesicle structures that stains for both SorLA and HSPA12A, while the green and red arrows point to more distally located vesicles positive only for either HSPA12A or SorLA, respectively. Scale bars represent 10 µm. The Western blots have been cropped to reduce the size of the figure. The entire membranes, without contrast adjustments, can be inspected in the Supplemental Materials.

HSPA12A and SorLA are proteins highly expressed in brain, kidney, testes and ovaries^32,33^. Thus, cells derived from either one of these tissues are apparent candidates to demonstrate co-localisation. Visualisation of the presumed co-localisation of HSPA12A and SorLA was assessed in primary cortical astrocytes prepared from p0 C57BL/6 mice. The astrocyte cultures were analysed by immunofluorescence microscopy using an antibody specific for the astroglia marker GFAP and found to very homogeneous all though a minor subset of the cells had very low expression of GFAP (Fig. 4a)^34^. The presence of both HSPA12A and SorLA in the primary astrocytes were verified by Western blotting of triton X-100 soluble and insoluble fractions prepared from astrocyte cultures suggesting a dual localisation (Fig. 4b). As exemplified with HSPA8 (Fig. 4b), the presence of HSP70 family members in both soluble and insoluble fractions is a recognised feature for several members of this family due to their dual cytosolic and nuclear localisation^35^.

SorLA was exclusively detected in the souble fraction, whereas Histone H3 was only detected in the insoluble fraction, disclosing a satisfactory (Fig. 4b). Immunofluorescence microscopy of cortical astrocyte cultures further demonstrated HSPA12A is located in the cytoplasm and nucleus (Fig. 4c). HSPA12A is associated with perinuclear compartments and vesicular-like structures throughout the cytoplasm of the cortical astrocyte cultures (Fig. 4c). Overlay analysis of SorLA and HSPA12A showed partial co-localisation of these proteins. In particular, SorLA and HSPA12A were found to co-localise in a fraction of perinuclear compartments/vesicles (Fig. 4c, white arrows). Co-localisation was almost absent in vesicles distally located to the nucleus, where SorLA and HSPA12A were mainly found in separate compartments (Fig. 4c, red and green arrows).

### HSPA12A alters the localisation of SorLA in HEK293 cells

**Figure 5:**
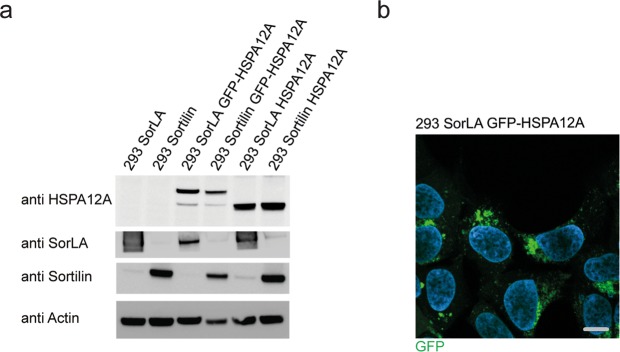
293 cell transfections and GFP-HSPA12A localisation. (**a**) Western blots showing the transfection of 293 cells with either SorLA or Sortilin and then double transfected with either HSPA12A or GFP-HSPA12A, as indicated. (**b**) GFP-HSPA12A transfected 293 cells shows that GFP-HSPA12A localises primarily to perinuclear compartments whereas as minor stanning in vesicles more distant to the nuclei are observed. The image displays a single frame obtained from a live cell recording and the scale bar represents 10 µm. The Western blots have been cropped to reduce the size of the figure. The entire membranes, without contrast adjustments, can be inspected in the Supplemental Materials.

**Figure 6:**
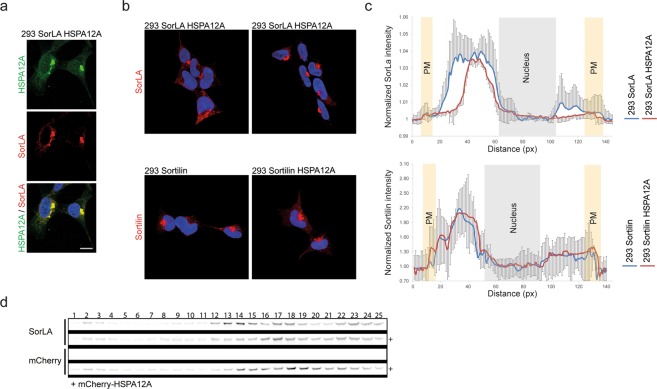
HSPA12A affects SorLA cellular distribution. (**a**) Immunofluorescence microscopy of HSPA12A (green) and SorLA (red) in 293 cells double transfected with SorLA and HSPA12A. (**b**) Immunofluorescence microscopy of SorLA (upper row) and Sortilin (lower row) in 293 transfected cells shows that SorLA, but not Sortilin, localisation is affected by HSPA12A. (**c**) Average intensity (±SEM) of SorLA (upper) and Sortilin (lower) across 293 SorLA or Sortilin single transfected cells (blue) and 293 cells double transfected with either SorLA or Sortilin and HSPA12A (red) (n = 18 (SorLA) or 10 (Sortilin) per transfection). (**d**) Western blot of SorLA in subcellular fractionation of endosomes by use of sucrose gradient centrifugation on lysate from 293 SorLA transfected cells and 293 cells double transfected with SorLA and mCherry-HSPA12A.

SorLA is an endocytic type-1 receptor engaged in intracellular transport of different proteins like lipoprotein lipase and GDNF^5,6^. Its short cd mediates the trafficking of SorLA due to binding with cytosolic adaptor proteins^7,8^. We hypothesised that HSPA12A binding to the cd of SorLA potentially could function as an adaptor protein and thereby alter the trafficking/localisation of SorLA. A series of stable single and double transfected HEK293 cell lines overexpressing SorLA, Sortilin and HSPA12A were established to address this eventuality. Cells were initially stably transfected with either SorLA or Sortilin, then the derived cell lines were stably transfected with wild-type HSPA12A (Fig. 5a). HEK293 cells and the derived SorLA-expressing cell line were in addition transfected with an N-terminal EGFP-tagged version of HSPA12A (Fig. 5b). Live imaging of GFP-HSPA12A transfected cells showed that GFP-HSPA12A localises similar to the HSPA12A pattern observed in the staining of cortical astrocytes, with the majority of staining present in large perinuclear vesicles and smaller fraction present in small mobile vesicles (Fig. 5b and Supplementary VIDEO). Immunofluorescence staining for SorLA and HSPA12A in double transfected cells revealed strong co-localisation in dense compartments close to the nucleus (Fig. 6a). More interestingly, the double transfectans also revealed a remarkable change in SorLA localisation compared to cells not transfected with HSPA12A (Fig. 6b). In accordance with previous reports, single transfected SorLA localised partly to larger perinuclear vesicles and partly to smaller vesicles throughout the cytoplasm^7^. When double transfected with HSPA12A, SorLA appeared highly constrained into perinuclear compartments, and very few distal SorLA-positive vesicles was observed (Fig. 6b). To verify this observation, the intensity of stained SorLA vesicles was quantified using ImageJ by placing a straight line passing the nucleus. The pixel intensity through this line resulted in information about the SorLA-positive vesicles’ distance from the nucleus, and if these vesicles are polarised. As observed by the eye, the presence of HSPA12A resulted in an accumulation of SorLA-positive structures close to the nucleus (Fig. 6c, red line), whereas SorLA-positive vesicles were more scattered in the absence of HSPA12A (Fig. 6c, blue line). Furthermore, SorLA-positive vesicles were polarised to one side of the nucleus with and without HSPA12A expression, but in the cells where HSAP12A were present, this SorLA polarisation was even more pronounced.

The changed subcellular distribution of SorLA was also demonstrated by subcellular fractionation of endosomes by use of sucrose gradient centrifugation. Here, the expression of HSPA12A resulted in the localisation of the majority of SorLA to denser vesicle (Fig. 6d). Since Sortilin and HSPA12A did not interact, Sortilin localisation was, as expected, not affected when double transfected with HSPA12A (Fig. 6b), suggesting that the altered SorLA distribution phenotype is caused directly by HSPA12A interaction with SorLA-cd.

### HSPA12A delays the internalisation of SorLA

**Figure 7:**
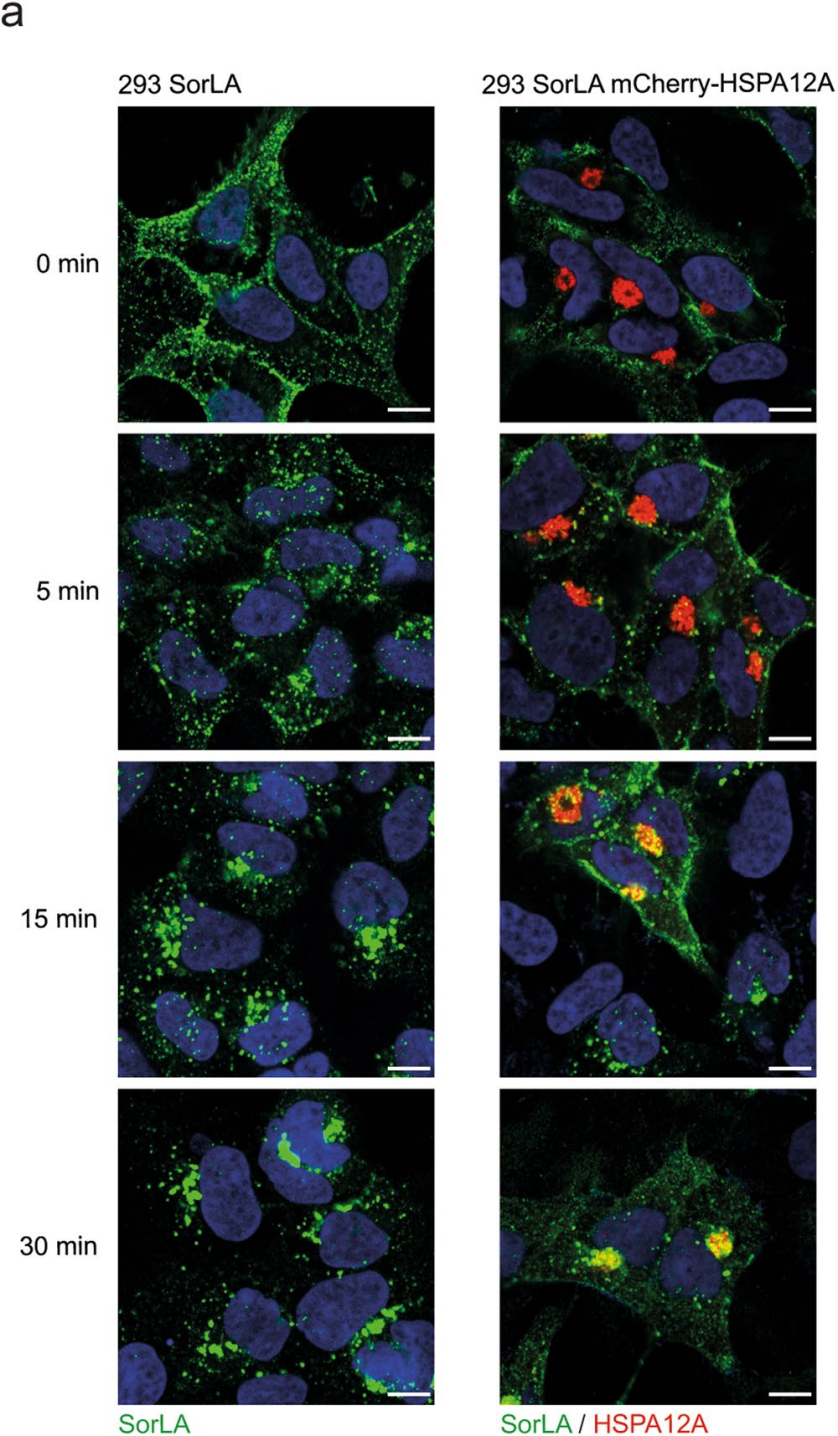
HSPA12A also affects SorLA internalisation. 293 SorLA transfected cells and 293 cells double transfected with SorLA and mCherry-HSPA12A were incubated with SorLA antibody and fixed at the indicated time points to evaluate SorLA internalisation.

It has previously been shown that SorLA is endocytosed from the surface within few minutes, in a process that involves AP-2^36^. To evaluate if HSPA12A influences the endocytosis of SorLA, living transfectans with SorLA and SorLA/mCherry-HSPA12A were saturated, on ice, with anti-SorLA antibodies on the surface membrane, where receptor endocytosis is arrested. By subsequent incubation at 37 °C and 5% CO_2_, receptor trafficking is revived, and the endocytic process and the following trafficking of SorLA were tracked by fixing and staining the cells at given time points (Fig. 7). As expected in single SorLA transfectants, the present of SorLA was almost absent at the surface after 5 min, and completely gone after 15 min. At these time points, perinuclear staining and some staining in well-defined vesicles distant from the nucleus were observed. In HSPA12A-positive cells, the endocytic process was evidently delayed, and clear surface staining up to at least 15 min was observed (Fig. 7, right panel). However, after 15 min of internalisation, a visual fraction of SorLA was also observed in the perinuclear area where HSPA12A is localised. After 30 min, most labelled SorLA was observed in HSPA12A-positive vesicles (Fig. 7). SorLA internalisation in SorLA/mCherry transfectans looked similar to SorLA internalisation in cells only transfected with SorLA (Supplementary Fig. 2). The data indicates that HSPA12A affects SorLA uptake and subcellular trafficking.

## Discussion

In this study, we searched for new cytosolic binding partners to SorLA, and identified HSPA12A as a novel adaptor protein. Importantly, we found that this binding is specific to SorLA, because no binding was observed to other members of the Vps10p-D receptor family. Endocytosis and subcellular localisation of SorLA was strongly affected by HSPA12A, which binds the cd of SorLA via two AC clusters in an ADP/ATP concentration dependent manner.

In the experimental setup, sequences obtained from a Y2H screen initially suggested the C-terminal of the heat shock protein HSPA12A as a potential cytosolic binding partner to the SorLA-cd. As Y2H screening assays are known to produce false positive signals, the interaction was verified using positive and negative bait plasmids. The positive control was the VHS domain from GGA2^27^ and an empty bait vector was the negative control (Fig. 1a). The interaction was verified by pull-down assays using full-length HSPA12A tagged with GST and a lysate from HEK293 cells overexpressing SorLA (Fig. 2a). Interestingly, no interaction was observed between Sortilin and other Vps10p-D receptors and other known retrograde transported receptors (MPR46 and MPR300) (Fig. 2b,c). Thus, it appears that HSPA12A binding is specific for SorLA, which could suggest that HSPA12A is an important factor for differences noted in subcellular localisation between SorLA and Sortilin^7^.

In the study, we found that HSPA12A shares important functional features with the conventional HSP70 proteins. As previously mentioned, heat shock proteins are characterised by an ADP dependent substrate binding activity. Since HSPA12A has a divergent nucleotide binding domain (compared to other HSP70), it has been questioned if HSPA12A should be categorised as a true member of HSP70 family^24^. Since SorLA-cd is the first identified substrate for HSPA12A, this study allowed us to test if ADP enhances HSPA12A substrate binding. From the pull-down experiment, we found that the interaction between HSPA12A and SorLA was influenced by the ATP/ADP level, with high concentration of ADP increasing the amount of SorLA precipitated by GST-HSPA12A, and low amount of ATP having the reversed effect (Fig. 2b). We conclude that the NBD in HSPA12A is functional and behaves similar to other HSPs regarding ADP dependent substrate binding. Based on this, we conclude that HSPA12A should be considered as a member of the HSP70 chaperone family^23^.

Truncated and point mutated constructs were used to identify the amino acids in SorLA-cd responsible for interacting with HSPA12A. Overall, binding of adaptor proteins to sorting receptors is mediated by linear motifs in the cd of the receptor, with the YxxØ and [DE]XXXL[LI] (where Ø indicates a bulky hydrophobic side chain) motifs as the canonical sequences facilitating binding to AP-1 and AP-2^7,30,31,37,38^. For SorLA, the pentameric acidic cluster (E34D35D36E37D38) is essential for endocytosis and for TGN to endosome trafficking (Fig. 3c). The C-terminal DDVPMVIA sequence interacts with the GGA’s, with impact for the anterograde transport^27^. Y2H analysis using truncated SorLA-cd mutants indicated that the binding to HSPA12A is dependent on an amino acid sequence harbouring 3 acidic clusters (G29-P50) (Fig. 3c). Mutating the 3 ACs independently into alanine residues, as well as amino acids in the vicinity of the C-terminal di-AC, revealed that the Y2H signals were downregulated by the pentameric AC and the C-terminal di-aspartic cluster (D47D48) mutants. The remaining mutants exhibited a response similar to the wild-type SorLA-cd. Accordingly, it is concluded that the 2 C-terminal acidic clusters both contributed to the binding between the SorLA-cd and HSPA12A. Thus, HSPA12A and GGA2 are sharing a C-terminal overlapping AC (D47D48) binding motif^27^. This could suggest that HSPA12A affects SorLA internalisation and subcellular trafficking by regulating binding of other adaptor proteins, simply by blocking their binding site, in particular GGA2 and AP-2.

From the HUGE protein database, SorLA and HSPA12A appear to co-express in several type of tissues, with significant expression in the brain and kidney^32^. This indicates that HSPA12A could have a physiological role for SorLA. To study the subcellular localisation of HSPA12A and potential co-localisation with SorLA, we used primary cortical mouse astrocytes. Western blots of soluble and insoluble fractions and IF demonstrated that HSPA12A, like other HSP70s, is present in both the cytosol and the nucleus, whereas SorLA, as expected, is only present in Triton soluble cytosolic fraction (Fig. 4b). Immunofluorescent labelling in these primary astrocytes further demonstrated that a fraction of the endogenous cytoplasmic-expressed HSPA12A displayed co-localisation with SorLA in vesicles in cortical astrocytes (Fig. 4c). To analyse if HSPA12A had an effect on SorLA in cells, or if the co-localisation was only a stochastic observation, stable cell lines expressing SorLA alone or together with HSPA12A were generated (Fig. 5a). Both high content screening and subcellular fractionation demonstrated a changed cellular localisation of SorLA when co-expressed with HSPA12A (Fig. 6a–c). We also observed that the endocytic capacity of SorLA was lowered by HSPA12A expression (Fig. 7). Together, these data clearly show HSPA12A has cellular effects on SorLA localisation and trafficking. Whether HSPA12A is a true adaptor protein for SorLA, or if it just blocks the binding of other adaptor proteins in given physiological situations, remains to be analysed. Since SorLA is important for recycling the insulin receptor from the endosome to the cell surface, HSPA12A expression could therefore be indirectly involved in the lack of insulin signalling in the brain^39^.

Heat shock protein, including HSP70, receives intensive attention among researchers, because it plays an important role in preventing protein misfolding and inhibiting aggregation, and therefore represents a class of proteins potentially involved in the pathogenesis of neurodegenerative diseases. This includes Alzheimer’s disease, where HSP70 has been demonstrated to have cytoprotective roles in inhibition of Aß oligomerisation and by enhancing the clearance of Aß^40,41^ As SorLA is the first described substrate for HSPA12A, cellular and physiological roles for HSPA12A are poorly elucidated. In a recent study investigating the functional effect of HSPA12A in ischemic brain injury, it was shown that *HSPA12A* knockout mice exhibited an enlarged infarct volume and aggravated neurological deficits compared to their wild-type littermates after stroke. Long-term examination revealed impaired motor function recovery and neurogenesis in the knockout mice, suggesting a neuroprotective role of HSPA12A in stroke patients^42^. Co-localisation of SorLA and HSPA12A is here only demonstrated to take place in cytoplasm. However, as SorLA is processed by alpha and presumably also by gamma secretase and thereby releasing the cd of SorLA, it is possible that HSPA12A could interfere with a putative SorLA-cd-mediated signalling in the nucleus^43–45^. Such regulations have previously been demonstrated for other receptors (e.g. ErB-4)^46^. Due to lack of specific antibodies detecting the cd of SorLA, these experiments have not been possible to perform.

Given the key role of SorLA in controlling neurotropic activity, e.g. by sorting of GDNF and BDNF^5,47^, and its major role in Alzheimer’s disease^12^, it is tempting to speculate if *HSPA12A* could also be a risk gene for Alzheimer’s or other psychiatric traits. *HSPA12A* is highly intolerant of loss-of-function variants (pLI = 0.98)^48^, suggesting that variants disrupting the function of *HSPA12A* loss of function^49^ are detrimental to humans. Interestingly, using data from a large exome sequencing study and aggregating all protein truncating variants (nonsense, frameshift, and essential splice site mutations) per gene, Satterstrom *et al*.^50^ recently identified an increased burden of LOF variants in *HSPA12A* in ADHD and autism cases. This finding is in-line with a previous study of post-mortem brains where the authors found reduced *HSPA12A* transcript expression in the prefrontal cortex of patients with schizophrenia^51^. Alteration of gene expression in the aging human brain may potentially influence the binding between HSAP12A and SorLA. The altered levels of competing ligands to the SorLA-cd and HSPA12A may be disease-related and contribute to progression of neurological disorders^49,52^.

To date, no genome-wide significant associations to disease have been identified with *HSPA12A*, which could be explained by the low number of known genetic variations in the gene, making it difficult to establish a statistically significant associations. In the large UK Biobank study, where around 500,000 individuals have been genotyped, there are only 2 known missense variants and no protein truncating variants (PTV) in the gene, making it difficult to pinpoint associations with specific phenotypes using conventional GWAS or PheWAS. (https://biobankengine.stanford.edu/gene/ENSG00000165868). As genome sequencing studies grow larger, it will become clear if rare variants in HSPA12A are part of the pathogenesis of Alzheimer’s or other psychiatric diseases.

Overall, we have identified HSPA12A as an adaptor that selectively binds to SorLA, which results in altered endocytosis and subcellular localisation. The finding of this specific chaperone can provide an explanation in the different cellular locations of SorLA and Sortilin. The study places the atypical HSPA12A as a member of the HSP70 family. The role of HSPA12A and its interplay with SorLA in the pathogenesis of psychiatric diseases remains to be determined.

## Materials and Methods

### Cell lines and transfections

293 human embryonic kidney cells (HEK293 cells) were cultured in Dulbecco’s Modified Eagle Medium (DMEM) (Invitrogen), supplemented with 10% fetal calf serum (Invitrogen) and P&S (10000 U/ml penicillin and 10 mg/ml streptomycin, Invitrogen).

SorLA and Sortilin transfected HEK293 cells have been described prior^7^. For creation of double transfected cells, full-length human HSPA12A was cloned into pIRESneo3 (Clontech) and pAcGFP1-C1 (Clontech) vectors. HEK293 SorLA and HEK293 Sortilin cells were transfected with these HSPA12A constructs using Fugene (Roche) transfection reagent, and stable double transfectants were subsequently selected with G418 (Invitrogen) and zeocin (Invitrogen).

### Preparation of astrocyte cell cultures

Brains of p0 C57BL/6 mice were surgically removed and placed in ice-cold sterile DPBS (Life-technologies). The meninges were peeled off and cortex tissue was isolated and carefully disrupted by puncturing before it was transferred to ice-cold DPBS. The cortex tissue was then sedimented, resuspended in DPBS with 0.25% Trypsin-EDTA (Sigma Aldrich) and incubated for 30 min at 37 °C. Digestion was stopped by 10% fetal calf serum and 2.4 units/ml DNase (Life Technologies). The digested tissue was sedimented at 200 × *g* for 5 min, the supernatant carefully discarded and the digested tissue were disrupted into single cells in DMEM by carefully pipetting up and down. The cells were sedimented at 300 × *g* for 5 min, resuspended in DMEM with 10% fetal calf serum and 100 μg/ml Primocin (Invitrogen) and transferred to Poly-L-lysine (Sigma Aldrich) coated cell culture flasks. After 48 hours, astrocytes were enriched by shaking of the cell flasks at 300 RPM at 37 °C for 3 hours and discarding all suspended cells afterwards. Enriched astrocytes were cultured for three to five days/one passage before used for experiments.

Mice used for isolation of astrocytes were bred and group-housed in the local animal facility at an ambient temperature and on a 12/12 h dark/light cycle (lights on 7 a.m.), under inspection of a veterinarian and according to Danish regulation for lab animals. The mice were euthanized before they were sacrificed in accordance with the international guidelines on the ethical use of animals (European Communities Council Directive of 24 November 1986; 86/609/EEC) and Danish guidelines. No ethical approval was needed to use tissues from euthanized animals, according to Danish law as stated by the Danish Ministry of Environment and Food (https://www.retsinformation.dk/Forms/R0710.aspx?id=162938), and no *in vivo* experiments on animal nor human material were used in these experiments.

### Pull-down analysis

Full-length human HSPA12A and GGA1 was cloned into the GST expression vector pGEX 4T-1 (GE Healthcare). Competent BL21 *E. coli* cells were transformed by heat shock with pGEX 4T-1 constructs and selected on 100 µg/ml ampicillin plates. Protein expression was induced by isopropyl-β-thiogalactoside (Sigma-Aldrich) to 1 mM final concentration, and GST fusion proteins were purified from the supernatant on Glutathione Sepharose 4B beads (GE Healthcare), according to the manufacturer’s protocol.

HEK293 cells, wild-type or transfectants with full-length SorLA, were lysed in ExB buffer (150 mM NaCl, 2 mM MgCl_2_, 2 mM CaCl_2_, 10 mM Hepes, pH 7.4) with 1% Triton X-100 and complete EDTA-free protease inhibitor for 15 min on ice. Afterwards, the solution was centrifuged at 15,682 × *g* for 10 min, and the supernatant was collected. For the pull-down experiment, supernatant was mixed with GST fusion protein pre-immobilised to Glutathione Sepharose 4B beads in Eppendorf tubes. ExB buffer was added as fill volume, and the mix was incubated overnight at 4 °C with slow rotation. Next day, beads were washed four times with ExB buffer, and proteins specifically immobilised on the beads were analysed by SDS-PAGE and Western blot. For pull-down with varying ATP/ADP concentrations, a pull-down solution containing beads with pre-immobilised GST fusion protein, HEK293 cell supernatant and ExB buffer was evenly split into aliquots. To these aliquots, adenosine 5′-triphosphate disodium salt hydrate (Sigma-Aldrich) or adenosine 5′-diphosphate sodium salt (Sigma-Aldrich) dissolved in ExB buffer was added before overnight incubation, for final concentrations of 0.1, 1.0 or 5.0 mM ATP/ADP. Next day, the aliquoted beads were washed, and proteins specifically immobilised on the beads was analysed by SDS-PAGE and Western blot.

### Constructs

Y2H library and the bait plasmids were purchased from Hybrigenics. The cd of SorLA K2160–A2214 (acc. NP_003096.1) was cloned into the Hybrigenics vector pB27 in fusion with LexA (http://www.hybrigenics.com). The SorLA di-alanine mutations were created by overlapping PCR reactions. The cd’s from Sortilin V778–E831 (acc. NP_002950), SORCS1B K1121-I1168 (acc. NP_443150.3), SORCS1C K1121-K1179 (P_001193498.1), SORCS2 K1101-S1159 (acc. NP_065828.2), SORCS3 K1145-V1122 (acc. NP_055793.1), BACE1 V477-K501 (acc. NP_036236.1), APP K593-N639 (acc. NP_001129601.1), MPR300 K2328-I2491 (acc. NP_000867.2) and MPR46 Q211-M277 (acc. NP_002346.1) were cloned into pB27. The purchased pP6 prey plasmids encoding HSPA12A and GGA2 were sequenced, revealing the following fragments: HSPA12A-T513-Y675 (acc. NP_079291.2) and GGA2-S37-N202 (acc. NP_055859.1). The HSPA12B-L370-N600 fragment (acc. NP_443202.3) was synthesised and cloned into the pP6 vector by Eurofins (Germany). Full-length HSPA12A (acc. NP_079291.2) was cloned into pIRESneo3 (Clontech). N-terminal GFP-tagged HSPA12A was expressed by inserting the coding sequence into the pAcGFP1-C1 vector (Clontech). N-terminal mCherry-tagged HSPA12A was expressed by exchanging the GFP with mCherry in the pAcGFP1-C1 construct. GST-tagged full-length HSPA12A and the GGA1 VHS domain (M1-S147) were expressed in *E. coli* BL21 using the pGEX4T1 vector (GE Healthcare).

### Y2H analysis

For transformation, pB27 and pP6 constructs obtained from Hybrigenics were transformed, and cells were amplified on plates selecting for both plasmids. Successfully transformed cells were suspended in sterile ddH2O and spot-plated (5 µl/spot) on plates prepared with 46 g/L yeast minimal agar SD base (Clontech) and 100 µg/ml P&S, and supplemented with either 640 mg/L Leu/Trp Dropout supplement (Clontech) for control plates or 640 mg/L Leu/Trp/His Dropout supplement (Clontech) for test plates. 3-aminotriazole (AT) (Sigma-Aldrich) was added to the plates to suppress bait auto-activation for SorLA-cd (10 or 15 mM) and MPR46-cd (20 mM).

### SDS-PAGE and Western blot

Samples were mixed with reducing sample buffer (50 mM Tris, 4% sodium dodecyl sulphate (SDS), 40% glycerol and 20 mM dithiothreitol), boiled for 4 min and run on NuPAGE 4–12% Bis-Tris Gels (Life Technologies) or custom made 8–16% SDS-PAGE gels. Western blotting was done according to standard protocols, using nitrocellulose membranes (Pharmacia-Amersham) and afterwards Tris-Buffered Saline, 0.1% Tween-20 and 5% non-fat milk as blocking agent. Primary antibodies (anti-SorLA (MABN1793, Sigma-Aldrich, Mab 20C11, 1:1000), anti-HSPA12A (HPA011273, Sigma-Aldrich, 1:1000), anti-HSP8A (sc-1059, Santa Cruz Biotechnology, 1:1000), anti-Histone H3 (ab1791, Abcam, 1:1000)) were diluted in blocking agent and incubated overnight at 4 °C. The following day, HRP-conjugated secondary antibodies (Abcam) were applied for 1 hour at room temperature. Visualisation was done in a LAS 3000 imager (Fujifilm) with Amersham ECL Western Blotting Detection Kit (GE Healthcare) as detection reagent.

### Immunofluorescence

Cells growing on coverslips were fixed in 4% formaldehyde followed by permeabilisation in PBS pH 7.4 with 0.25% Saponin (Sigma-Aldrich). Primary antibodies (anti-SorLA (MABN1793, Sigma-Aldrich, Mab 20C11, 1:100), anti-HSPA12A (HPA011273, Sigma-Aldrich, 1:50), anti-Sortilin (MABN1792, Sigma-Aldrich, Mab F11, 1:100)) in PBS pH 7.4 0.25% Saponin were incubated for 2 hours at room temperature. Secondary labelling was performed with Alexa-Fluor-488 and -568-conjugated antibodies (Invitrogen) at 1:350 dilution, and nuclei staining was finally performed with 1 μg/ml Hoechst 33258 (Invitrogen). Coverslips were mounted with Fluorescence Mounting Medium (Dako). Stained cells were analysed using a LSM780 laser-scanning confocal microscope (Carl Zeiss) using a 63x C-Apochromat water immersion objective with an NA of 1.2. Image capturing and post-analysis were done with the Zen software (Carl Zeiss).

### Quantifying SorLA cellular distribution

Immunofluorescence staining of SorLA in HEK293 SorLA and HEK293 SorLA HSPA12A transfected cells were analysed in ImageJ. Single cells with a comparable cross section length were identified, and an intensity profile going through the centre of the nucleus and the SorLA-positive Golgi/ER perinuclear complex was calculated. Averaging of intensity profiles across cells was done by fitting the profile of each cell using the first signal from the SorLA-positive cell membrane on the Golgi/ER side.

### Subcellular fractioning of astrocytes

Crude cell lysate was obtained by lysis of astrocytes in a 1% Triton X-100, 150 mM NaCl and 50 mM Tris pH 7.4 lysis buffer. The lysate was centrifuged at 15,682 × *g* for 10 min at 4 °C, and the supernatant was collected (soluble fraction). The pellet was washed twice in lysis buffer and resuspended directly in reducing SDS sample buffer then boiled for 10 min (insoluble fraction, containing nuclei, mitochondria, and a small insoluble fraction of the plasma membrane (dense lipid rafts)).

### Subcellular fractionation and sucrose step gradient

Stably transfected HEK 293 expressing SorLA alone or together with HSPA12A-mCherry were washed in ice cold Tris buffered saline (TBS) containing 0.4 mM phenylmethylsulfonyl fluoride (PMSF), resuspended in 800 µl homogenisation buffer (250 mM sucrose, 10 mM HEPES-KOH (pH 7.2), 1 mM EDTA (pH 7.5), 1 mM MgOAc and 250 mM PMSF and disrupted by five passages through a 21-gauge needle followed by eight passages through a metal cell cracker with 9 µm gap. Post-nuclear supernatants were subjected to velocity gradient centrifugation (SW41Ti/Beckmann, 77000 × g 18 min) using a continuous sucrose gradient (0.3–1.2 M sucrose) prepared by mixing on a BioComp gradient master (45°/10 min and 80°/1 min). Twelve fractions of 1 ml were collected using a BioComp piston gradient fractionator. Fractions 7–9 were pooled and further separated by overnight equilibrium gradient centrifugation (SW41Ti/Beckmann, 77000 × g) on a sucrose step gradient (0.8–1.2 M). One ml fractions were collected and analysed by Western blotting.

### Statistics

One-way ANOVA followed by post-hoc Tukey multiple comparison test was used for Fig. 2c. Significance was assumed at p < 0.05.

## Supplementary information

Supplementary figures
