# Supplementary material for: HSPA12A targets the cytoplasmic domain and affects the trafficking of the Amyloid Precursor Protein receptor SorLA
Source: Sci Rep. 2019 Jan 24;9:611. doi: 10.1038/s41598-018-37336-6 (PMC6345817; doi:10.1038/s41598-018-37336-6)
Supplement: Supplementary file 1 — Supplementary figures [file 41598_2018_37336_MOESM1_ESM.pdf]

HSPA12A targets the cytoplasmic domain and affect the trafficking of the Amyloid Precursor Protein receptor SorLA

Peder Madsen*[1], Toke Jost Isaksen[1], Piotr Siupka[1], Andrea E. Tóth[1], Mette Nyegaard[1], Camilla Gustafsen[1], Morten S. Nielsen*[1]

[1], Department of Biomedicine, Aarhus University, 8000 Aarhus, Denmark

*Corresponding author: Morten S. Nielsen (mn@biomed.au.dk) and Peder Madsen (pm@biomed.au.dk), Ole Worms Allé 3, 8000 Aarhus, Denmark.

Running head: HSPA12A affect SorLA trafficking

Supplementary Material

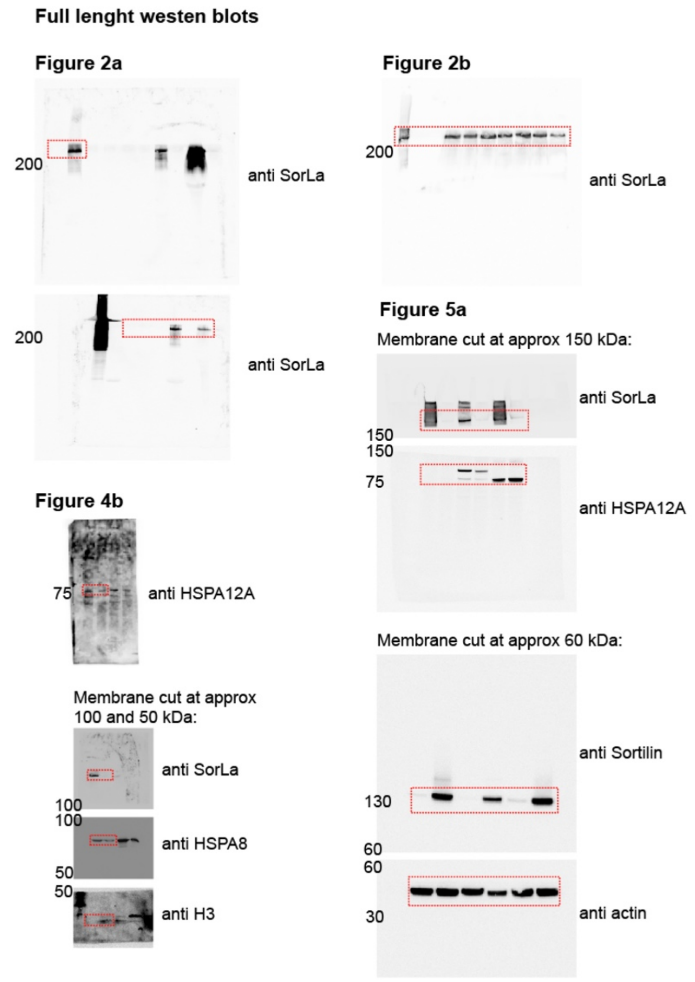

Supplementary figure 1
Displayed are the original uncropped membranes form the Western blot used in figure 2, 4 and 5 in the main manuscript. The red line highlights the cropped area. For membrane in figure 4 and 5, the membranes have been divided after blotting, but before adding antibodies in order to demonstrated the presence of different proteins in the same cell lysate and in the same loading.
All blots have visualized using in a LAS 3000 imager (Fujifilm) with Amersham ECL Western Blotting Detection Kit (GE Healthcare) as detection reagent.
No blots have been stitched together from different membranes.

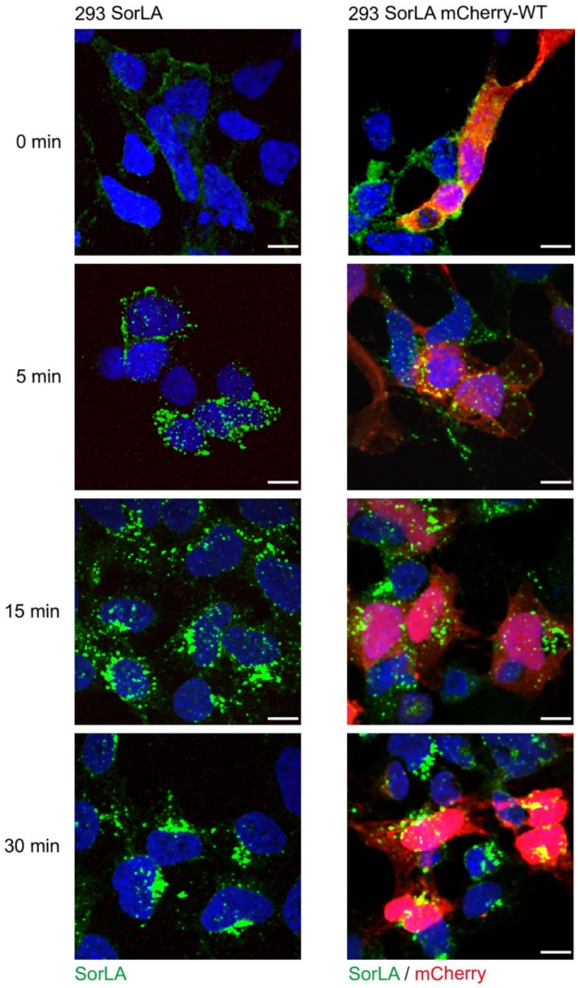

Supplementary figure 2
293 SorLA transfected cells and 293 cells double transfected with SorLA and mCherry were incubated with SorLA antibody and fixated at the indicated time points to evaluate if mCherry alone affect SorLA internalization.
Cells were fixed in 4% formaldehyde followed by permeabilization in PBS pH 7.4 with 0.25% Saponin (Sigma-Aldrich). Primary antibodies (anti SorLA (MABN1793, Sigma-Aldrich, Mab 20C11, 1:100) in PBS pH 7.4 0.25% Saponin, were incubated for 2 hours at room temperature. Secondary labelling was performed with Alexa-Fluor-488 and -568-conjugated antibodies (Invitrogen) 1:350 dilution and nuclei staining was finally performed with 1 µg/mL Hoechst 33258 (Invitrogen).
Stained cells were analyzed using an LSM780 laser-scanning confocal microscope (Carl Zeiss) using a 63× C-Apochromat water immersion objective with an NA of 1.2. Image capturing and post analyzing were done with the Zen software (Carl Zeiss).
